# Assessment of multi-professional primary healthcare center quality by patients with multimorbidity

**DOI:** 10.1186/s12913-024-11315-2

**Published:** 2024-08-20

**Authors:** Antoine Dany, Paul Aujoulat, Floriane Colin, Jean-Yves Le Reste, Delphine Le Goff

**Affiliations:** 1 ER 7479 SPURBO, University of Western Brittany, Brest, France; 2 Department of Human Sciences, University of Western Brittany, Brest, France; 3 Department of General Practice, University of Western Brittany, Brest, France

**Keywords:** Multimorbidity, Primary health care, Quality of health care, Patient satisfaction, Face validity

## Abstract

**Background:**

The main aim of this study was to build an item bank for assessing the care quality of multi-professional healthcare centers (MPHCC) from the perspective of patients with multimorbidity. This study was part of the QUALSOPRIM (QUALité des SOins PRIMaires; primary healthcare quality) research project to create a psychometrically robust self-administered questionnaire to assess healthcare quality.

**Methods:**

First, twelve experts built an item bank using data from a previous qualitative work and a systematic literature review. Second, the validity of each item was assessed in a sample of patients. Adult patients with multimorbidity were recruited from six French MPHCC. Items were assessed based on ceiling effects, the level of missing or neutral responses and patient feedback. Patient feedback was recorded after the item bank completion. Based on results, items were validated, improved, or removed during expert meetings. In case of disagreement the Delphi method was used to reach consensus.

**Results:**

The study sample included 209 outpatients. The most frequent medical conditions were cardiovascular risk factors, cardiovascular diseases and rheumatological conditions. In total, a bank of 109 items classified in nine domains was built. The validity assessment led to the removal of 34 items. Retained items explored a variety of topics related to care quality: availability, accessibility, premises’ layout and building, technical care, expertise, organization, relationships with caregivers and communication, involvement and personal relationships.

**Conclusions:**

This study allowed cross-validation of a bank of 75 items, leading to a complete picture of the patient perception of care quality items. Overall, patients were generally satisfied with their care at the MPHCC. Nonetheless, there were still numerous items on subjects for which patients’ satisfaction could be improved.

**Supplementary Information:**

The online version contains supplementary material available at 10.1186/s12913-024-11315-2.

## Introduction

Patients with multimorbidity have one chronic disease and at least another (acute or chronic) disease, a biopsychosocial risk factor and/or a somatic risk factor. They often experience complex healthcare interactions [1]. To meet their increasing care needs, healthcare systems are shifting to a more patient-centered and comprehensive approach with increasing numbers of multi-professional healthcare centers (MPHCC) [2–5]. Patient-centered care, including user experience, quality of care and outcomes, can help to produce high-quality healthcare systems [6]. Healthcare quality can be measured to improve patient-centered care and healthcare quality [7, 8].

Primary healthcare provides integrated, accessible healthcare services by clinicians who address most healthcare needs by developing a partnership with patients, and by practicing in the context of family and community [9]. Healthcare quality can be evaluated from the patients and healthcare professionals’ (HCP) perspective. Independent medical evaluation is the gold standard approach to assess healthcare quality from the HCP perspective. To assess healthcare quality from the patients’ perspective, two approaches can be used: (i) patient experience that reflects the patients’ perception of the received care, and (ii) patient satisfaction that reflects the gap between the quality of the received care and the personal expectations [10, 11]. The patient perspective allows assessing the patient centeredness of care, which is a feature of high-quality care, like safety and efficacy [12].

A recent systematic review revealed that many self-assessment instruments are available to measure care quality at MPHCC from the perspective of patients with multimorbidity. These instruments capture many patient experiences, but few have strong psychometric properties. This review highlighted the need of a valid, responsive, reliable and robust instrument to assess and improve the quality of primary care [13]. Therefore, the aim of the QUALSOPRIM (QUALité des SOins PRIMaires; primary healthcare quality) project was to build a new evaluation tool with robust psychometric properties. First, a qualitative study was performed using in-depth, face-to-face interviews with 26 patients, 23 informal caregivers and 57 HCPs from five MPHCC in France. This study showed that patients, informal caregivers, and HCPs shared a common vision to improve primary care quality. Nine core domains for care quality were identified [14].

The main aim of the present study was to build an item bank for MPHCC care quality assessment by patients with multimorbidity.

## Methods

### Item bank construction

Twelve experts [two general practitioners (GP), a psychometrician, and seven GP trainees] developed the bank of items that were formulated according to the Question Appraisal System-1999 guideline [15]. Response options were phrased during expert meetings. To formulate the response options experts were instructed to assign an objective quantity to each response option (e.g. numbers or common situations that patients could easily relate to).Item response options were designed to ensure the possible range of responses would be captured. The maximum number of response options was set at five.

The item bank included questions on patient experience and satisfaction (available as supplementary file). As healthcare quality is a multi-dimensional concept, items were classified into several domains on the basis of previous phases of the QUALSOPRIM project, clinicians’ experience at a MPHCC and psychometric model requirements.

### Validity assessment of the item bank

Validity assessment was conducted in Brittany, a region in Northwest France. It started in the summer 2019 and was planned to last 1 year. The assessment goal was to validate, improve or remove items. Face validity and psychometric dysfunction were assessed to reduce the item bank size. Indeed, reducing the item bank size will facilitate all subsequent work.

#### Inclusion criteria

Patients (≥ 18 years of age) with at least two chronic conditions, and followed by at least two HCPs were recruited. Purposive sampling was used to ensure that enough patients had home care and an informal caregiver so that related items could be assessed. At least 25% of recruited patients needed to have an informal caregiver and 50% were to have home care to assess corresponding items. An investigating physician identified the eligible patients at the MPHCC. Patients were included if they were able to express their informed consent and signed a written consent. Patients were anonymized and received an ID number. The experimental protocol was approved by the ethical research committee (Comités de Protection des Personnes SUD-EST IV) and was categorized as observational.

#### Procedures

GP trainees underwent training on the item bank construction methodology and the required administrative tasks. A GP trainee met the included patients at their MPHCC or their home to explain how to assess the item bank. Patient data were collected: age, sex, and care details (duration, frequency, HCP type, place of care), current medical conditions. Each patient completed a paper version of the item bank. The GP trainee could provide help for the first ten items, if needed. Patients with an informal caregiver could complete the related domain items with their help. Each answer was scored from 1 (total disagreement) to 4 (total agreement); neutral answers (unconcerned or did not mind) were scored zero. Following the item bank assessment completion, patients participated in an open discussion with the GP trainee who included them in the study. The aim was to evaluate how well they understood the item bank and to detect problematic items (e.g. unknown words or irrelevant answers). Patients were encouraged to be uncompromisingly honest and to highlight all difficulties, inconsistencies, or misunderstandings. Patients could suggest improvements and judge the overall item bank acceptability.

Patient feedback was recorded in an Excel spreadsheet and was used to propose adaptations or improvements for each item in a meeting with the same expert group that constructed the item bank. In case of disagreement during these meetings, the Delphi method was later used to reach a consensus (> 80% agreement), one to four rounds were planned [16].

The scores for each item were recorded manually and independently in two Excel spreadsheets by two GP trainees. The two spreadsheets were automatically compared to identify mismatches. For each mismatch, the two GP trainees entered the definitive value in a third spreadsheet, after discussion. After the last validation by all investigators, the final data file was frozen.

Items were considered to have a poor face validity if they had either a low response rate (missing answer rate > 10%) or relevant negative patient feedback. Items were considered to have psychometric dysfunction if they had either a high percentage (> 75%) of neutral/unconcerned responses or a floor/ceiling effect (> 90% of most negative or most positive answers).

Items with negative feedback, poor face validity and/or psychometric dysfunction were improved if possible or removed from the item bank.

## Results

**Fig. 1:**
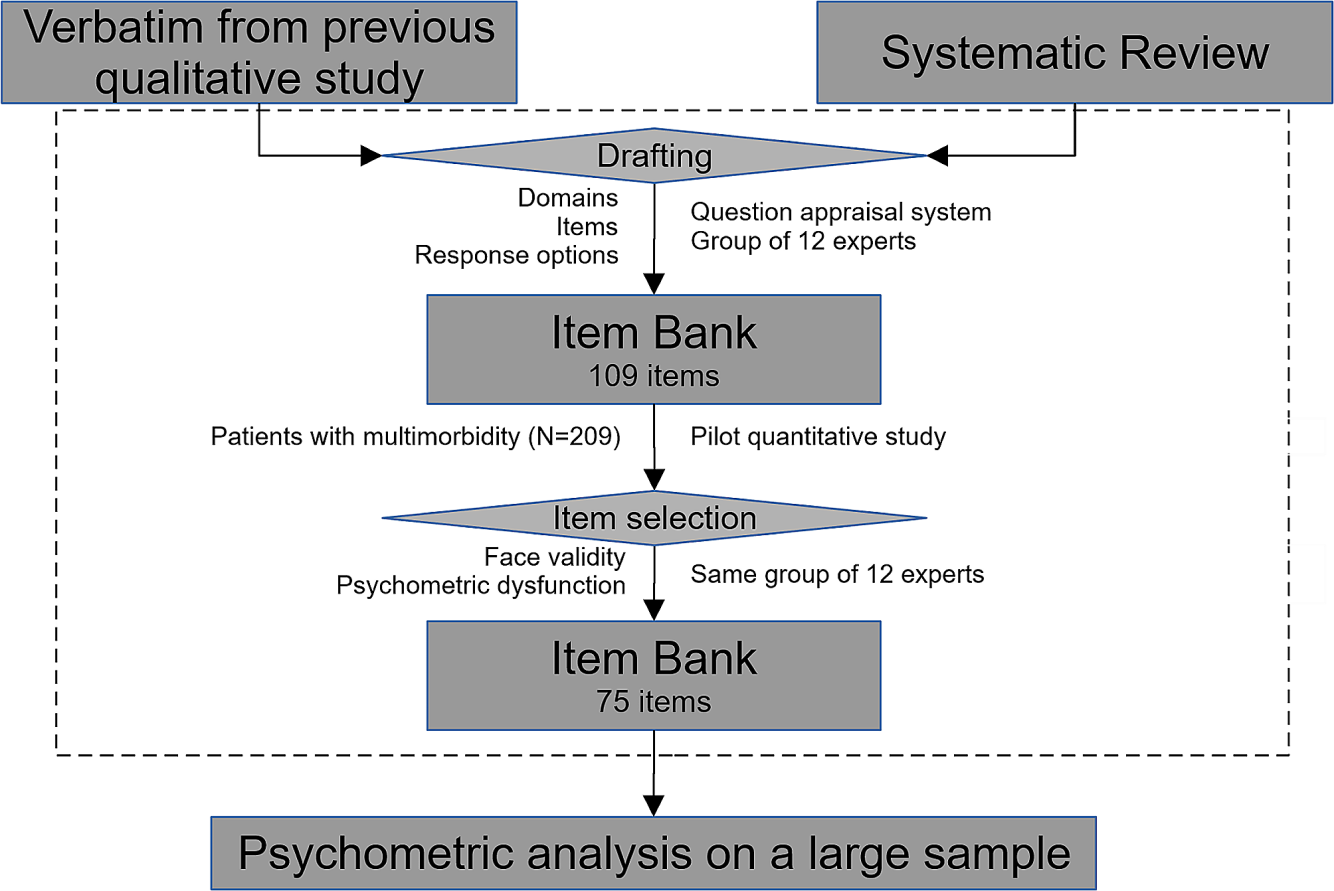
The main results of the present paper are within the dashed rectangle. Results from previous QUALSOPRIM studies used in the present paper are above it. The next phase of the QUALSOPRIM project is below. QUALSOPRIM: QUALité des SOins PRIMaires; primary healthcare quality

The main results and their integration in the whole research project are presented in Fig. 1.

### Item bank construction

#### Item drafting

In total, 109 items were included in the item bank in the form of a self-report questionnaire. Each item was given 3 to 5 ordered response categories (Likert scale).

#### Classification in domains

The initial version of the item bank had ten domains: nine core domains and a general satisfaction domain. The nine core domains were: (i) “HCP availability”, (ii) “Care accessibility”, (iii) “MPHCC layout”, (iv) “Medical-technical care”, (v) “GP’s expertise”, (vi) “Care organization within the MPHCC”, (vii) “Patient-HCP relationship and communication”, (viii) “Patient’s role in their care”, and (ix) “Main informal caregiver’s role in the care pathway”. Each domain included 6 to 18 items. The general satisfaction domain included three items and was at the end of the item bank. It will serve as a point of comparison for later-stage psychometric assessments.

The item bank is available as supplementary material.

### Validity assessment of the item bank

In total, 209 patients (60% of women) were included at six MPHCC from July 26, 2019 to October 29, 2020. Their characteristics are presented in Table 1. Their age ranged from 29 to 99 years (median = 74 years) and was between 67 and 83 years in 53% of patients. Almost half of patients had been followed by their current GP for > 10 years, exclusively at the MPHCC or at home and at the MPHCC. Most patients were seen every 3 months, mainly by their GP and nurses. The most frequent medical conditions were cardiovascular risk factors, cardiovascular diseases and rheumatological conditions.

**Table 1:** Patient characteristics

Variable *n*_total_=209	*n* or mean	% or ±SD
**Sex**		
Male	85	40.67
Female	124	59.33
**Age**	73.8	±12
**Follow-up duration**		
< 1 year	7	3.35
1-5 years	63	30.14
5-10 years	45	21.53
> 10 years	94	44.98
**Follow-up frequency**		
Several times per month	10	4.78
Once per month	68	32.54
Once every 3 months	117	55.98
Once every 6 months	14	6.70
**Follow-up location**		
Home exclusively	24	11.48
MPHCC exclusively	95	45.45
Both	90	43.06
**HCP type consulted (besides physicians)**		
Nurse	162	77.88
Physiotherapist	66	31.73
Podiatrist	62	29.95
Dentist	42	20.10
Others	15	7.25
**Condition type**		
Cardiovascular risk factors	143	68.42
Cardiovascular diseases	89	42.58
Rheumatological conditions	91	43.54
Respiratory diseases	34	16.27
Cancer	35	16.75
Thyroid conditions	28	13.40
Other chronic conditions*	26	12.44
Psychiatric conditions	23	11.00
Neurological conditions	18	8.61
Digestive diseases	21	10.05
Ophthalmological conditions	17	8.13
Hematological conditions	11	5.26
Kidney diseases	7	3.35

n: number; SD: standard deviation; MPHCC: multi-professional healthcare centers; HPC: healthcare professionals; *Complex regional pain syndrome, Dermatological problems, Sarcoidosis, ENT

The time needed to complete the item bank assessment ranged between 45 min and 1 h. Both patients and GP trainees repeatedly stressed that as the item bank was very long, it was difficult to maintain concentration and questions were missed. Therefore, several items and answers were simplified, and terminology was changed to improve clarity. The important terms in each item were underlined. Neutral answers were added for some items to avoid missing answers when patients felt unconcerned/indifferent. The layout was improved to facilitate reading. Overall, 69% of items were retained, resulting in an adapted 75-item bank. Domain-specific results are summarized in Table 2. Thirty-four items had psychometric dysfunction and/or poor face validity. They were removed from the item bank. For detailed results see the additional table provided as supplementary material.

**Table 2:** Item selection results

Retained items	Removed items
**HCP availability**	
1. Ease to get a consultation slot or a home visit 2. Possibility to get a regular consultation 3. Possibility to get an urgent consultation 4. Waiting time before a scheduled consultation 5. Waiting time before an unscheduled consultation 6. Possibility to get a regular home visit 7. Possibility to get an urgent home visit 8. Frequency of consultations with the GP 9. Continuity of care at the MPHCC 10. Information on care continuity at the MPHCC	1. Ease to get a consultation slot suited to the patient’s schedule 2. Possibility to change HCP at the MPHCC 3. Possibility to group several appointments together
**Care accessibility**	
1. Accessibility for people with reduced mobility 2. Layout suitability for people with reduced mobility 3. Possibility to reach an HCP directly 4. Possibility to text the GP	1. MPHCC location 2. MPHCC signage 3. Consultation cost 4. Car parking possibility 5. MPHCC accessibility by public transport 6. Possibility of third-party payment (partial or total)
**MPHCC layout**	
1. Overall comfort 2. Building sound insulation 3. Entertainment in the waiting room 4. Information displayed in the waiting room	1. Building temperature 2. Building ventilation 3. Building sound insulation for medical confidentiality 4. Interior layout 5. Layout suitability for discretion 6. Premises’ cleanliness
**Medical-technical care**	
1. Treatment adaptation by GP 2. Exam room hygiene 3. Pain management during nursing care 4. Hygiene precaution during nursing care 5. Allied health professionals’ compliance with the medical prescriptions 6. Allied health professionals’ adaptation of medical prescriptions 7. MPHCC medical equipment	1. GP’s examination 2. HCPs’ hand hygiene 3. Motivation and encouragement from physiotherapists
**GP’s expertise**	
1. GP’s responsiveness to difficult health problems 2. GP’s capacity to self-questioning 3. Regular general health monitoring 4. GP’s way of delivering exam results 5. Adaptation of the consultation length to the situation	1. Adequate focus of the consultation on the problem that led to the consultation
**Care organization within the MPHCC**	
1. Having the same GP in the long term 2. Having the same allied health professionals for long time 3. Presence of a substitute physician in the absence of the usual GP 4. Effective use of the medical record 5. Dialogue between HCPs for complex situations 6. Information sharing among HCPs after a small health problem 7. Physician’s ability to seek advice from colleagues 8. Diversity of HCPs within the structure 9. Access to specialist physician consultations	1. Quality of care by substitute physicians 2. Communication effectiveness between 3. HCPs for home care 4. Information transmission from the secretariat to HCPs 5. Possibility to refuse the presence of a student during care
**Patient-HCP relationship and communication**	
1. Reception by the secretariat 2. Patient understanding when communicating with HCPs 3. Relationship closeness with caregivers 4. Trust in HCPs 5. Listening by HCPs 6. Patient capacity to express without fear of being judged 7. Physician’s non-avoidance of awkward topics 8. Disturbances, such as phone calls during the consultation 9. Possibility to fully explain all worrying issues 10. How much did the patient understand at the consultation end	1. Appropriateness of secretary’s requests for medical information 2. Medical record sharing 3. GP’s openness on complementary medicine
**Patient’s role in their care**	
1. Capacity to inform the patients about their disease 2. Capacity to inform the patients about treatment side effects 3. Capacity to inform the patients about their eligibility for supports or accommodation programs 4. Psychological support offer 5. HCPs’ reassurance 6. HCP team’s capacity in helping to overcome a loss of morale 7. HCPs’ encouragement 8. HCPs’ capacity to let the patient actively participate in important decision-making 9. HCPs’ capacity to let the patient actively participate in the medical follow-up	1. Capacity of focus groups to provide additional knowledge on the disease 2. Interest in therapeutic education sessions 3. Possibility to join a discussion group 4. Drafting advanced care directives
**Main informal caregiver’s role in the care pathway**	
1. Capacity to keep the informal caregiver informed 2. Possibility for the informal caregiver to ask questions 3. HCP responsiveness to solicitations by the informal caregiver 4. Informal caregiver’s involvement in the medical follow-up 5. Informal caregiver’s involvement in important decision 6. Informal caregiver’s opportunity to participate in care 7. HCP capacity to make the informal caregiver feel comfortable in their role 8. HCP team’s capacity to adequately relieve the informal caregiver for daily tasks 9. HCP team’s monitoring of the informal caregiver’s fatigue 10. HCP team’s monitoring of the informal caregiver’s psychological state 11. HCP team’s efforts to reduce the difficulties encountered by the informal caregiver at home 12. Home equipment to help the informal caregiver 13. Care adaptation in function of the informal caregiver’s schedule 14. Solutions when the informal caregiver is unavailable	1. Informal caregiver’s involvement expected by the HCP team 2. Resources dedicated to informal caregivers at the MPHCC 3. Impact of the informal caregiver’s role defined by the HCP team on the patient-informal caregiver relationship 4. Adaptation of schedules and of task distribution to relieve the informal caregiver

MPHCC: multi-professional healthcare centers; HCP: healthcare professionals; GP: general practitioner

## Discussion

Overall, 75 items of the item bank had suitable validity for future development. Despite our purposive sampling technique, the study sample adequately represented the rural and semi-rural French population demographics. Indeed, most patients were women (60%) and in the 60–80 years age group (56%), in line with French national epidemiological data [17]. The patient feedback and quantitative results on validity allowed cross-validation, leading to a complete picture of the patient perception of care quality items. A satisfactory outcome from this study was that overall, patients were generally satisfied with care at the MPHCC. This is supported by the fact that low rated items were infrequent, the presence of many ceiling effects and the absence of any floor effect. Although many items on subjects considered important by patients had to be removed because of ceiling effects, there were still numerous items for which patients’ satisfactions could be improved which is essential for the future questionnaire.

Almost all items assessing HCP availability had proper validity. This probably come from the fact that they raised important issues for many patients in the context of growing primary care needs (e.g. choosing or changing GP). Indeed, the region has experienced a decline in HCP numbers. For instance, the number of GP in Brittany decreased by 5.6% from 2012 to 2021 [18]. The situation was probably more significantly affected in the rural and semi-urban areas that participated to this study. Removed items were on subsidiary matters which may depend very much on the situation. For example, most patients were not concerned about the possibility of grouping appointments with different HCPs, possibly because the chosen MPHCC were small with a limited number of HCPs. However, this item may be relevant in larger MPHCC.

In the Care accessibility domain, the four items that showed acceptable validity were about two subtopics: accessibility for people with reduced mobility and ease to reach HCP. Although building compliance with accessibility for people with reduced mobility is mandatory in France, there were still few cases where exemptions or delay had been granted. Ease to reach GP was a frequent expectation for patients with multimorbidity which was emphasized by the increasing number of modalities available through mobile phone, and internet software. This was however often perceived as overburdening by HCP who wanted to be seldom contacted by patients outside consultation. The items on consultation costs and receiving care without upfront payment were both removed because of ceiling effects. These items were not relevant for the French healthcare system where patients with multimorbidity receive care without upfront costs and 100% of their medical expenses are covered by the national health insurance system. However, these items can be important in other countries where patients can face high costs [19]. Moreover, as economic pressure on the French healthcare system increases, they might become relevant also in France.

Few items on the MPHCC layout and building were considered important by patients. They were related to overall comfort, building sound insulation, things to pass time in the waiting room and information displayed in the waiting room. Conversely, there were strong ceiling effects for several items (e.g., building temperature, ventilation) which probably reflected the little interest by patients in these factors. Indeed, although some MPHCC included in this study were not built recently and their layout was not always optimal and ergonomic, these were not a priority for patients.

Concerning the medical-technical and GP’s expertise domains, although most patients felt they had little ability or legitimacy to judge these aspects, most items had proper validity. The ceiling effect on the GP’s capacity to focus on the health condition that led to the consultation may be related to the fact that patient-centered care is one of the main GP’s skills [20, 21]. Most patients rated highly their GP’s skills. This is consistent with data from the National French Directorate of Research, Studies, Evaluation and Statistics (DREES; Direction de la Recherche, des Études, de l’Évaluation et des Statistiques) showing that 88% of French people are satisfied with the quality of care and information provided by the GP on their health status [22].

Most items on care organization within the MPHCC had proper validity. For instance, the item on consultations with specialists had the lowest score, possibly due to the scarcity of some specialists in MPHCC in France. Recently, this issue has slightly improved because young specialists are more attracted by interprofessional cooperation in primary care centers, besides their role in hospitals. For instance, in the Finistère department, three MPHCC offer consultations with a cardiologist, neurosurgeon, orthopedic surgeon, psychiatrist, gastroenterologist, urologist, and dermatologist. This item is thus particularly valuable for the item bank because it contained a large number of high rated items and lacked low-rated items.

Items on the Patient-HCP relationship and communication showed adequate face validity. Only over-specific items such as medical record sharing, communications among professionals, and openness on complementary medicine were removed because of poor validity.

The validity of most items on the patient’s role in their care domain are promising because patient involvement in their care (e.g. therapeutic patient education) will be developed at these MPHCC in the coming years [23]. Again, only over-specific items assessing formal intervention such as focus group or dedicated therapeutic education session were removed because of poor validity. Indeed, patients were not familiar with these expert terms or participated in therapeutic education in a non-formal way. Interestingly, although they were introduced in the French public health code in 2005 most patients (62.21%) said that they had never discussed about advanced care directives with their HCPs. This indicator needs to be measured to facilitate approaching this topic because HCPs must support patients in drafting their advanced care directives [24, 25].

Most items on the main informal caregiver’s role were hard to evaluate due to the smaller amount of available data (i) by design (only 25% of recruited patients had an informal caregiver), and (ii) because this domain was at the questionnaire end and thus patients might have lost interest. Thus, for conservative reasons, very few items were removed from this domain compared with the other domains. The results for the item on informal caregiver resources at the MPHCC showed that 68% of patients reported that few or no resources were available for the informal caregiver. This confirms the poor informal caregivers’ recognition in France as well as in other developed countries [26, 27].

The present study had two important limitations. First, the length of the item bank negatively affected the study feasibility. Indeed, almost all patients lost motivation and became disinterested towards the questionnaire end. The adapted version with fewer items should address this major limitation. Second, as this was a pilot study, the sample size was small. Therefore, only important item psychometric dysfunctions were detected and items with poor psychometric properties might still be present.

The next study phase will analyze the item bank psychometric properties using the item response theory [28, 29] in a larger patient sample. This will allow further reducing the item number and assessing the domain structure and psychometric properties.

## Conclusion

A bank of 109 items was built and then reduced to 75 items on the basis of their validity assessed in a sample of 209 patients. Retained items explored a large variety of topics related to care quality: availability, accessibility, premises’ layout and building, technical care, expertise, organization, relationships with caregivers and communication, involvement and personal relationships. The analysis of item validity provided a valuable insight on how patients with multimorbidity evaluated their MPHCC care. Most removed items were about topics that were either subsidiary (e.g., possibility to refuse the presence of a student during care), fully satisfactory (e.g., consultation cost), over specific (e.g., possibility to group several appointments together) or which patients felt unable to answer (e.g., communication effectiveness between professionals for home care).

### Electronic supplementary material

Below is the link to the electronic supplementary material.

Supplementary Material 1

Supplementary Material 2

## Data Availability

The datasets used and/or analysed during the current study are available from the corresponding author on reasonable request.

## Abbreviations

DREES: National French Directorate of Research, Studies, Evaluation and Statistics
GP: General practitioners
HCP: Healthcare professionals
DREES: National French Directorate of Research, Studies, Evaluation and Statistics
MPHCC: Multi-professional healthcare centers
QUALSOPRIM: QUALité des SOins PRIMaires (primary healthcare quality)
