# Supplementary material for: Assessment of multi-professional primary healthcare center quality by patients with multimorbidity
Source: BMC Health Serv Res. 2024 Aug 20;24:954. doi: 10.1186/s12913-024-11315-2 (PMC11337593; doi:10.1186/s12913-024-11315-2)
Supplement: Supplementary file 1 — Supplementary Material 1 [file 12913_2024_11315_MOESM1_ESM.docx]

**Qualsoprim pilot study**

**Date: Place: Patient #:**

# Healthcare professionals’ availability

- 1. **Can you easily obtain an appointment for a visit? (at the healthcare centre or home visit)**

easily

4

quite easily

3

not very easily

2

with difficulty

1

- 1. **How would you rate the waiting time to obtain an appointment for a non-urgent visit?**

reasonable

4

quite reasonable

3

rather unreasonable

2

unreasonable

1

- 1. **How would do you rate the waiting time to obtain an appointment for an urgent visit? (acute health problem)**

reasonable

3

quite reasonable

2

1

unreasonable

not concerned

0

- 1. **How would you rate the waiting time for a scheduled visit?**

satisfactory

4

rather satisfactory

3

somewhat unsatisfactory

2

unsatisfactory

1

not concerned

0

- 1. **How would you rate the waiting time for a walk-in visit?**

satisfactory

4

rather satisfactory

3

somewhat unsatisfactory

2

unsatisfactory

1

not concerned

0

- 1. **How would you rate the waiting time to obtain an appointment for a non-urgent home visit?**

reasonable

4

quite reasonable

3

21

rather unreasonable

unreasonable

1

not concerned

0

- 1. **How would you rate the waiting time to obtain an appointment for an urgent home visit? (acute health problem)?**

reasonable

4

quite reasonable

3

21

rather unreasonable

unreasonable

1

not concerned

0

- 1. **Are you satisfied with the frequency with which you are able to consult your referring doctor?**

no problem

3

21

sometimes troubled

often troubled

1

not concerned

0

- 1. **Does the healthcare centre's permanence of care seem sufficient to you? (time slots, opening days, weekends and holidays)**

sufficient

3

21

sometimes insufficient

insufficient

1

- 1. **Is the information on care continuity correctly** **transmitted to you? (information sheets or orally, etc.)**

well transmitted

4

rather well transmitted

3

21

rather poorly transmitted

poorly transmitted

1

# Care accessibility

- 1. **Is access to the structure suitable for people with reduced mobility? (parking spaces, access ramp, orientation strips, etc.)?**

adapted

4

rather adapted

3

insufficiently adapted

2

not adapted

1

not concerned

0

- 1. **Is the layout of the structure suitable for people with reduced mobility? (door width/elevator/suitable space, etc.)**

adapted

4

rather adapted

3

insufficiently adapted

2

not adapted

1

not concerned

0

- 1. **When you wish to talk to a healthcare professional, can you reach them directly?**

easily

3

quite easily

2

1

with difficulty

not concerned

0

- 1. **Can you communicate with your doctor by email or text message?**

yes

2

no but I wish I could

12

it does not interest me

0

# Healthcare centre layout

- 1. **Is the healthcare centre generally furnished in a comfortable manner?**

comfortable

4

rather comfortable

3

rather uncomfortable

2

uncomfortable

1

- 1. **Is the healthcare centre well insulated from noisy elements?**

well insulated

3

some noises

2

noisy

1

- 1. **Are you satisfied with what is offered in the waiting room? (magazines, music, books, children's toys, etc.)**

many options

3

some missing

23

almost none

1

it does not interest me

01

- 1. **Is information displayed in the waiting room relevant?**

lot of relevant information

3

few relevant information

2

no relevant information

1

it does not interest me

0

# Nursing and non-physician care

- 1. **Does your doctor adapt your treatment as your disease progresses? (e.g. modification of the treatment/dose, home care services)**

as soon as required

4

my doctor is sometimes slow in doing it

3

my doctor is often slow in doing it

2

he rarely if ever does it

1

- 1. **What do you think about exam room hygiene? (disinfection of equipment between patients, use of disposable equipment, etc.)**

clean

4

rather clean

3

rather dirty

2

dirty

1

- 1. **Do nurses take enough precautions to limit pain during your care?**

they are very attentive

4

they are quite attentive

3

they sometimes lack attention

2

they barely pay attention

1

I do not need any nursing care

0

- 1. **Do nurses follow sufficiently the hygiene standards during your care?**

they always pay attention

4

they rather pay attentive

3

they sometimes overlook it

2

they barely pay attention

1

I do not need any nursing care

0

- 1. **Are you satisfied with the non-physician staff's adherence to medical prescriptions?**

satisfied

4

rather satisfied

3

somewhat unsatisfied

2

unsatisfied

1

I do not need non-physician care

0

- 1. **Are you satisfied with the non-physician staffs’ ability to adapt to changes in prescriptions? (in function of how your health status changes; for instance, dressing adapted to the different stages of a wound)**

satisfied

4

rather satisfied

3

somewhat unsatisfied

2

unsatisfied

1

I do not need non-physician care

0

- 1. **What do you think of the structure's medical equipment (electrocardiogram, physiotherapy equipment, etc.)**

numerous and rather good quality materials

3

some missing or obsolete equipment

2

essential equipment is missing

1

the equipment is not a quality criterion for me

0

# Skills of the general practitioner

- 1. **Is your doctor reactive enough when you have health problems for which it is difficult to make a diagnosis? (e.g. prescription of additional tests, referral to specialists)**

reactive

4

quite reactive

3

not very reactive

2

very unreactive

1

- 1. **Does your doctor accept to reassess/call into question a diagnosis when you consult several times for the same health problem without any improvement?**

my doctor is willing to reassess the diagnosis or treatment strategy

3

sometimes I have to insist on being heard

2

they often stick to their position

1

not concerned

0

- 1. **Are you satisfied with the regularity of your health follow-up in general? (e.g. screening, blood tests, changes in your chronic disease)**

my follow-up is regular and of good quality

4

my follow-up is rather regular

3

my follow-up is rather irregular

2

my follow-up is irregular and of poor quality

1

- 1. **Are you satisfied with how your doctor explains to you the results of your exams? (e.g. blood tests, X-ray exams)**

satisfied

4

rather satisfied

3

rather unsatisfied

2

unsatisfied

1

- 1. **Does your doctor sufficiently adapt the consultation time in function of the health problem that led you to book the visit?**

flexible within reason

3

fairly/quite flexible

2

not very flexible

1

# Care organization within the MPHCC

- 1. **Are you able to be followed by the same referring doctor over the long term?**

easily

3

rather hardly

2

hardly or impossible

1

- 1. **For your regular care, are you always followed by the same team of healthcare professionals (non-physician)?**

most of the time

4

rather often

3

rather seldom

2

seldom

1

I do not need non-physician care

0

- 1. **If your referring doctor is absent, another doctor can receive you in the event of an urgent problem? (other doctor in the centre or replacement doctor)**

most of the time

4

rather often

3

sometimes

2

seldom

1

- 1. **When you see doctors, do you think that they make effective use of your health record? (e.g. your history, allergies)**

most of the time

4

rather often

3

sometimes

2

seldom or never

1

- 1. **Do the healthcare professionals consult each other when dealing with complex situations? (e.g. clinical, social, etc.)**

they formally meet for decision making

2

they work independently or exchange ideas informally

1

I do not know

0

- 1. **In the event of a minor health problem, do healthcare professionals share rapidly the required information?**

rapidly

4

quite rapidly

3

rather slowly

2

slowly, it is mainly through me that they have the information

1

I do not know

0

- 1. **Do you think that your doctor will easily contact a colleague to ask for an opinion in the event of doubts about your care?**

readily seeks advice if in doubt

3

sometimes delays asking a colleague for advice

2

they do not question themselves or do not dare to ask for an opinion

1

not concerned

0

- 1. **Do you think that the centre provides a sufficiently wide range of caregivers? (podiatrist, midwife, etc.)?**

there is everything you need

3

I would add one or two professions

2

several important professions are missing

1

- 1. **Does the centre facilitate access to specialist doctor consultations?**

there is a sufficient range of specialists accessible on site

3

certain specialties that I need are not accessible on site

2

one generally has to go outside (hospital, clinic, etc.)

1

# Healthcare professional-patient relationship and communication

- 1. **Do you receive a good welcome from the secretariat? (smile, politeness, respect, etc.)**

pleasant

4

rather pleasant

3

rather unpleasant

2

unpleasant

1

- 1. **Do you think that communication with the healthcare professionals at the healthcare centre is adapted to your level of understanding?**

adapted

4

rather adapted

3

insufficiently adapted

2

not adapted

1

- 1. **Are you satisfied with the relational proximity that you can have with your healthcare professionals?**

satisfied, they know how to set the right distance

4

quite satisfied

3

rather unsatisfied

2

unsatisfied

1

- 1. **Do the healthcare professionals inspire trust/confidence?**

I feel fully trustful

4

I feel quite trustful

3

I'm rather distrustful

2

I do not trust them

1

- 1. **During consultations, do you think that the healthcare professionals are listening to you?**

fully

4

quite well

3

rather insufficiently

2

insufficiently

1

- 1. **Do you feel free to express yourself without fear of remarks from the healthcare professionals?**

fully free

4

quite free

3

rather holding back

2

really holding back

1

- 1. **When necessary, does your doctor ask you questions about topics you consider embarrassing? (intimate subjects, convictions, spirituality, etc.)**

they appropriately approach uncomfortable subjects

3

they sometimes avoid awkward topics

2

they often do not discuss or avoid uncomfortable topics

1

there has never been any need to bring up any awkward topics

0

- 1. **Do you ever get disturbed during your consultation, whether by the telephone, the secretariat or otherwise?**

never or rarely

4

sometimes

3

often

2

most of the time

1

- 1. **Have you ever left a consultation without having been able to clearly explain what was worrying you?**

never or rarely

4

sometimes

3

often

2

most of the time

1

- 1. **Did it happen already that you left a consultation without understanding what the healthcare professional(s) had told you?**

never or rarely

4

sometimes

3

often

2

most of the time

1

# The patient’s role in care (or: in the care pathway)

- 1. **Have you been adequately informed about your disease? (e.g. prognosis, possible treatments, physical consequences)**

I am very well informed

4

I am reasonably well informed

3

I am not very well informed

2

I received very little information and/or I have to find out myself

1

- 1. **Have the possible side effects of your treatment been sufficiently explained to you by your caregivers?**

clear explanations

4

rather clear

3

rather confusing

2

No explanation or very confusing

1

- 1. **Do caregivers inform you of the supports or adaptations available to you in relation to your chronic illness? (meal delivery, social assistance, parking pass, etc.)**

I am very well informed

4

I am reasonably well informed

3

I am not very well informed

2

I received very little information although I am interested

1

I am not interested

0

- 1. **Are your satisfied with the proposed psychological support?**

fully satisfied

4

rather satisfied

3

rather unsatisfied

2

unsatisfied

1

I do not feel concerned

0

- 1. **Do healthcare professionals reassure you throughout your disease?**

reassuring, they always have a comforting word to say

4

rather reassuring

3

rather not reassuring

2

not reassuring

1

- 1. **When you are feeling low, are you satisfied with how the healthcare team helps you to get through the ordeal? (e.g. medications, moral support)**

satisfied

4

quite satisfied

3

rather unsatisfied

2

unsatisfied

1

not concerned

0

- 1. **Do healthcare professionals encourage you throughout your disease? (e.g. diet, following your treatment)**

most of the time

4

often

3

sometimes

2

seldom or never

1

- 1. **Do healthcare professionals allow you to play a sufficiently active role in planning your future? (e.g. staying at home, moving to a retirement home)**

I feel fully involved

4

I feel moderately involved

3

I feel little involved

2

I have no choice in anything

1

not concerned

0

- 1. **Do you feel that you are in charge of your care and do you make decisions about your health together with the healthcare professionals?**

I feel fully involved

4

I feel moderately involved

3

I feel little involved

2

I have no choice in anything

1

# The main informal caregiver’s place in the care pathway

- 1. **Do you have an informal caregiver: a person who helps you in your daily life to cope with your health condition?**

yes

1

0 no ⇒ go to section J

- 1. **Is your informal caregiver kept informed of the progress of your condition and its treatment?**

most of the time

4

often

3

sometimes

2

seldom or never

1

- 1. **Do healthcare professionals ensure that your informal caregiver can ask questions?**

most of the time

4

often

3

sometimes

2

seldom or never

1

- 1. **Are the healthcare professionals at the healthcare centre receptive to your informal caregiver’s requests? (e.g. reachable by the informal caregiver)**

receptive

4

rather receptive

3

rather resistant

2

resistant

1

- 1. **Do healthcare professionals involve your informal caregiver in understanding your disease and its management?**

most of the time

4

often

3

sometimes

2

seldom or never

1

- 1. **Do healthcare professionals involve your informal caregiver in planning your future? (e.g. end of life, moving to an adapted structure)**

my informal caregiver is always involved in the decisions concerning my future

4

often

3

sometimes

2

seldom or never

1

- 1. **Do caregivers provide opportunities for your informal caregiver to participate in your care?**

most of the time

4

often

3

sometimes

2

seldom or never

1

no treatment seems eligible to me

0

- 1. **Do you think that the healthcare professionals make your informal caregiver feel comfortable in their role?**

quite a lot

4

moderately

3

a little

2

very little

1

- 1. **Concerning the daily tasks related to your health condition, does the healthcare professional team relieve sufficiently your informal caregiver?**

quite a lot

4

moderately

3

a little

2

very little

1

- 1. **Do healthcare professionals monitor your informal caregiver’s fatigue level?**

most of the time

4

often

3

sometimes

2

seldom or never

1

- 1. **Do healthcare professionals monitor your informal caregiver’s psychological state?**

most of the time

4

often

3

sometimes

2

seldom or never

1

- 1. **Do healthcare professionals try to reduce the difficulties that your informal caregiver encounters at home? (e.g. adapting the house, proposing help)**

most of the time

4

often

3

sometimes

2

seldom or never

1

- 1. **Does the equipment in your home facilitates you informal caregiver’s daily life? (e.g. patient lifter, nursing bed, commode chair)**

quite a lot

4

moderately

3

a little

2

very little

1

- 1. **Is your care adapted to your informal caregiver’s schedule in order to have as little impact on them as possible?**

most of the time

4

often

3

sometimes

2

seldom or never

1

- 1. **When your informal caregiver is not available (vacation, illness, etc.) are you offered solutions to replace them?**

the relay is perfectly assured

4

the relay is partially assured

3

the relay is poorly assured

2

the relay is poorly or not assured

# Overall satisfaction

- 1. **Today, do you think that your health has improved or that you are living well despite your health problems? (e.g. constraints, pain, regular care)**

I feel well, stabilised, and less in pain

4

I feel quite well, stabilised, and a little less in pain

3

my disease is always a burden, but I feel supported and well cared for

2

I am not satisfied with my health state and my disease course

1

- 1. **Are you satisfied with your care at the healthcare centre?**

satisfied

4

quite satisfied

3

rather unsatisfied

2

unsatisfied

- 1. **Are you satisfied with the home healthcare teams?**

satisfied

4

quite satisfied

3

rather unsatisfied

2

unsatisfied

not concerned

0
