# Supplementary material for: Assessment of multi-professional primary healthcare center quality by patients with multimorbidity
Source: BMC Health Serv Res. 2024 Aug 20;24:954. doi: 10.1186/s12913-024-11315-2 (PMC11337593; doi:10.1186/s12913-024-11315-2)
Supplement: Supplementary file 2 — Supplementary Material 2 [file 12913_2024_11315_MOESM2_ESM.docx]

| **Ceiling effects (% of positive answers)** | **Poor discrimination power** |
| --- | --- |
| **HCP availability.** | |
| - Ease to obtain a consultation appointment adapted to the patient's schedule (95) | - Possibility to change HCP within the MPHCC - Opportunity to group several appointments together |
| **Care accessibility** | |
| - MPHCC location (89%) - MPHCC signage (91%) - Consultation cost (96%) | - Car parking availability - MPHCC accessibility by public transport - Possibility of third-party payment (partial or total) |
| **MPHCC layout** | |
| - Building temperature (93%) - Ventilation (94%) - Sound insulation for confidentiality (91%) | - Interior layout - Layout suitability for discretion - Building cleanliness |
| **Medical-technical care** | |
| - GP’s examination (96%) - HCPs’ hand hygiene (89%) | - Motivation and encouragement by physiotherapists |
| **GP’s expertise** | |
| - whether the consultation was adequately focused on the problem that led to that visit (97%) | Ø |
| **Care organization within the MPHCC** | |
| Ø | - Care quality by substitute physicians - Effectiveness of the communication means between HCPs for home care - Information transmission from the secretariat to HCPs - Possibility to refuse the presence of a student during care |
| **Patient-HCP relationship and communication** | |
| - Inappropriate requests of medical information by the secretary (90%). | - Medical record sharing - GP’s openness on complementary medicine |

| **Ceiling effects (% of positive answers)** | **Poor discrimination power** |
| --- | --- |
| **Patient’s role in their care** | |
| - Do focus groups provided additional knowledge about the disease (89%) | - Patient’s interest in therapeutic education sessions - Possibility of joining a discussion group - End-of-life instructions |
| **Informal caregiver’s role in the care pathway** | |
| - whether the place given to the carer by the HCP team negatively influenced the patient-carer relationship (97%). | - Presence of carer-dedicated resources at the MPHCC - Place given to the carer by the HCP team - Schedule and task distribution adaptations to relieve the carer |
